# Hippocampal TNF-α Signaling Mediates Heroin Withdrawal-Enhanced Fear Learning and Withdrawal-Induced Weight Loss

**DOI:** 10.1007/s12035-021-02322-z

**Published:** 2021-02-13

**Authors:** Shveta V. Parekh, Jacqueline E. Paniccia, Lydia O. Adams, Donald T. Lysle

**Affiliations:** Department of Psychology and Neuroscience, University of North Carolina at Chapel Hill, CB#3270, Chapel Hill, NC 27599-3270 USA

**Keywords:** Opioid, Withdrawal, Neuroimmune, TNF-α, PTSD, Cytokines

## Abstract

There is significant comorbidity of opioid use disorder (OUD) and post-traumatic stress disorder (PTSD) in clinical populations. However, the neurobiological mechanisms underlying the relationship between chronic opioid use and withdrawal and development of PTSD are poorly understood. Our previous work identified that chronic escalating heroin administration and withdrawal can produce enhanced fear learning, an animal model of hyperarousal, and is associated with an increase in dorsal hippocampal (DH) interleukin-1β (IL-1β). However, other cytokines, such as TNF-α, work synergistically with IL-1β and may have a role in the development of enhanced fear learning. Based on both translational rodent and clinical studies, TNF-α has been implicated in hyperarousal states of PTSD, and has an established role in hippocampal-dependent learning and memory. The first set of experiments tested the hypothesis that chronic heroin administration followed by withdrawal is capable of inducing alterations in DH TNF-α expression. The second set of experiments examined whether DH TNF-α expression is functionally relevant to the development of enhanced fear learning. We identified an increase of TNF-α immunoreactivity and positive cells at 0, 24, and 48 h into withdrawal in the dentate gyrus DH subregion. Interestingly, intra-DH infusions of etanercept (TNF-α inhibitor) 0, 24, and 48 h into heroin withdrawal prevented the development of enhanced fear learning and mitigated withdrawal-induced weight loss. Overall, these findings provide insight into the role of TNF-α in opioid withdrawal and the development of anxiety disorders such as PTSD.

## Introduction

Post-traumatic stress disorder (PTSD), a devastating mental illness, is highly comorbid with substance use disorder (SUD), as nearly 40% of individuals diagnosed with PTSD are diagnosed with SUD [1]. Specifically, opioid abuse has one of the highest prevalence rates of any comorbid SUD. It has been reported that 33.2% of individuals with an opioid use disorder (OUD) currently meet criteria for comorbid PTSD and 41% of those have a lifetime history of PTSD [2, 3]. Notably, comorbidity estimates of heroin use disorder and PTSD are as high as 66% [4], and this has substantial clinical consequences. Co-occurring PTSD in heroin use disorder is associated with an earlier addiction onset age, longer addiction durations, higher rates of attempted suicide, and poorer occupational functioning [4]. Consequently, the mechanisms surrounding this comorbidity present an important area of research in order to improve the treatment, recovery, and outcomes of clinical populations with these psychopathologies.

Heroin use and withdrawal may give rise to long-term neurobiological changes believed to underlie major symptoms of PTSD, such as the fear learning, hyperarousal, and/or re-experiencing events, and increased vulnerability future stressors. Opioid-dependent patients display increased distress on the perceived stress scale, and interestingly, abnormally high cortisol levels have been correlated with an individual’s increased discontinuation risk for recovery [5]. Consistent with this, patients undergoing opioid withdrawal have markedly elevated salivary cortisol levels [6, 7]. These withdrawal-induced physiological effects are pronounced during opioid withdrawal and can have long-lasting consequences [8]. Within the preclinical literature, opioids also have been shown to increase the production of corticotropin-releasing factor mRNA [9], and plasma corticosterone levels in rats [10]. The elevated cortisol or corticotropin-releasing factor response associated with opioid use and withdrawal has prominent immune consequences, as it is related to altered cytokine expression in both human and animal models [11–13] and observed across multiple anxiety disorders, such as PTSD [14–17].

Our laboratory has previously shown that dorsal hippocampal (DH) interleukin-1 (IL-1) signaling is responsible for the development of stress-enhanced fear learning (SEFL), a reliable and reproducible animal model of fear-related features of PTSD [18]. Although it is difficult to incorporate all the symptoms of PTSD into a preclinical animal model, SEFL effectively demonstrates hyperarousal and greater susceptibility to future fear learning, a prominent component of human PTSD. In the SEFL paradigm, rats previously exposed to a severe stressor (inescapable foot shocks) show an exaggerated or enhanced fear response to a mild form of stress in a separate, distinct context. The hyperarousal and enhanced reactivity response captured using the enhanced fear learning paradigm offers the opportunity to investigate an important, critical symptom of clinical PTSD. Our laboratory has shown that exposure to the severe stressor in this model induces a time-dependent, region-specific increase in interleukin-1β (IL-1β) immunoreactivity within the dentate gyrus (DG) of the DH [19]. This stress-induced increase in IL-1β is causally related to enhanced fear learning, as blockade of IL-1 signaling with IL-1 receptor antagonist (IL-1RA) following the severe stressor prevented the development of SEFL [20]. These data suggest that alterations in hippocampal neuroimmune signaling directly lead to maladaptive behavioral responses, such as enhanced fear learning and increased sensitivity to future stressors.

We have recently developed an animal model for chronic escalating heroin administration and withdrawal that we use in combination with the enhanced fear learning paradigm [21]. This model produces reliable withdrawal behaviors such as wet dog shakes, diarrhea, and teeth chattering 24 h after the last heroin dose. Importantly, this model also produces significant weight loss in heroin-treated animals, a hallmark sign of rodent opioid withdrawal. Strikingly, this model of heroin administration and withdrawal is capable of producing enhanced fear learning and long-lasting hyperarousal [21]. This exciting finding suggests that prolonged opioid exposure and subsequent withdrawal elicit increased stress vulnerability and produce persistent hyperreactivity in a rodent model. In addition to the behavioral consequences, heroin withdrawal induces a similar region-specific increase in IL-1β immunoreactivity within the DG [21]. Critically, intra-DH IL-1RA during heroin withdrawal prevented the development of heroin withdrawal-enhanced fear learning [21]. These studies indicate that the altered DH IL-1 signaling during heroin withdrawal produces long-lasting neuroadaptations that result in exaggerated fear learning behavior.

Although our research has primarily focused on IL-1β, central cytokines function in concert to facilitate learning processes [22], and there is evidence that IL-1β does not independently influence heroin withdrawal and enhanced fear learning. Other proinflammatory cytokines, such as TNF-α, are critical in learning processes [23], and alter the response to anxiety and distress. Interestingly, the literature suggests that TNF-α and IL-1β have a synergistic relationship in multiple inflammatory mechanisms [24, 25], as both cytokines stimulate proinflammatory responses and increase cytokine production. This suggests that the actions of TNF-α and IL-1β are interconnected; however, little is known about the role TNF-α plays in opioid use and withdrawal, as well as the development of hyperarousal states of PTSD. TNF-α has been shown to be upregulated following opioid use [26] and during withdrawal [27]. Likewise, translational evidence from both rodent and clinical studies has implicated TNF-α in stress-related disorders, such as PTSD [28–30]. Moreover, TNF-α has an integral role in learning and memory processes, as well as cognitive functioning, suggesting a possible role in fear learning behaviors [31–34]. Collectively, these studies support the idea that TNF-α signaling facilitates the ability of chronic heroin administration and withdrawal to enhance future fear learning.

The current studies test the hypothesis that chronic heroin and withdrawal are capable of inducing alterations in TNF-α expression in the DH, and that this DH TNF-α expression is functionally relevant to the development of future enhanced fear learning and withdrawal symptoms, as indicated by weight loss. To this end, experiment 1 determined the consequence of chronic heroin administration and withdrawal on TNF-α expression in the DH. Our analysis focused on the DH as this region has been shown to be critical to context-dependent fear learning and conditioning [35–37]. Specifically, we focused on the DG subregion, as this is where we observed increased IL-1β expression during heroin withdrawal [21]. We identified increased TNF-α immunoreactivity within the DG following chronic heroin administration 0, 24, and 48 h into withdrawal. Subsequently, experiment 2 tested whether blocking DH TNF-α signaling with etanercept, a TNF-α inhibitor, 0, 24, and 48 h into heroin withdrawal prevented the development of enhanced fear learning and withdrawal-induced weight loss. We show that etanercept significantly attenuated enhanced fear learning, as well as mitigated withdrawal-induced weight loss. Together, these experiments are the first to test whether DH TNF-α signaling following chronic heroin administration and withdrawal is critical to the development of enhanced future fear learning and mediates other withdrawal-related detriments, such as weight loss.

## Methods and Materials

### Animals

Adult male Sprague Dawley rats (225–250 g, Charles River Laboratories, Raleigh, NC) were individually housed under a reversed 12-h light-dark cycle. Rats were given ad libitum access to food and water, and were regularly handled throughout experimentation. All procedures were conducted with approval from the University of North Carolina at Chapel Hill Institutional Animal Care and Use Committee.

### Drug Administration

Heroin (diacetylmorphine hydrochloride, National Institute on Drug Abuse (NIDA) Drug Supply Program, Bethesda, MD, USA) was dissolved in sterile 0.9% saline to produce 1.0, 2.5, 5.0, 7.5, or 10.0-mg/mL solutions and stored at 4°C until time of injection.

### Surgery and Infusion Delivery

For stereotaxic surgery, animals were anesthetized with a 1.0-mL/kg intraperitoneal injection of 9:1 (vol/vol) ketamine hydrochloride (100 mg/mL) mixed with xylazine (100 mg/mL). Guide cannulae (26 Gauge, Plastics One, 324 Roanoke, VA, USA) were directed bilaterally at the DH 325 (AP − 3.4 mm, ML ± 3.1 mm, DV − 2.2 mm, 15°, relative to bregma). Animals were given 2 weeks for postoperative recovery prior to the start of experimental procedures. Animals were randomly assigned to receive heroin or saline in the chronic escalating heroin administration and then either a saline or etanercept infusion treatment. Etanercept (Millipore Sigma, St. Louis, MO, USA), a TNF-α inhibitor, was dissolved in sterile saline (2.5 μg/μL). Animals underwent the chronic heroin escalating administration and were given three infusions of etanercept or saline at 0, 24, and 48 h into withdrawal (Standard Infuse/Withdraw PHD 2000 Infusion Syringe Pump, Harvard Apparatus, Holliston, MA, USA). Forty-eight hours prior to the first infusion, animals were given a sham microinjection to allow for habituation to the injection experience. Animals were microinfused with 1.25 μg of etanercept or saline vehicle per hemisphere at a rate of 0.25 μL/min, and the injectors were left in place for 1 min to allow for drug diffusion away from the injection site.

### Tissue Collection and Histology

In experiment 1, animals were sacrificed by transcardial perfusion 0, 24, 48, and 72 h into withdrawal. Animals that were perfused 1h following their last heroin injection were classified to be in the 0-h withdrawal group, as they were considered to have heroin in their system and, therefore, had not undergone withdrawal. Animals perfused at 24 h following the last injection are considered to be in the 24-h withdrawal group, while the same applies for the animals perfused at 48h and 72h groups respectively. Animals were terminally anesthetized with 9:1 (vol/vol) ketamine hydrochloride (100 mg/mL) mixed with xylazine (100 mg/mL), and transcardially perfused with ice-cold phosphate buffer (PB; pH = 7.4) followed by 4% paraformaldehyde in 0.1 M PB. Brains were extracted and post-fixed in 4% paraformaldehyde for 6 h, and used 30% sucrose for cryoprotection with 0.1% sodium azide at 4 °C. Once the brains were saturated with sucrose, brains were cut into 40-μm coronal sections on a cryostat (Leica CM 3050 S, Leica Microsystems, Buffalo Grove, IL, USA). For experiment 2, animals were sacrificed by rapid cervical dislocation and DH cannula placement was verified.

### Immunohistochemistry

Experiment 1 used fluorescent immunohistochemistry (IHC) to examine alterations in DH TNF-α in the DG. The IHC protocol used here has been described previously [19, 21]. Briefly, tissue sections were washed three times for 10 min in 0.1M phosphate buffer (PB, pH = 7.4), followed by a 1-h incubation in 5% normal goat serum (NGS) and 0.5% TritonX100 in 0.1M PB at room temperature. Tissue was incubated in primary antibody:rabbit anti-TNF alpha (1:1000, Abcam, Cambridge, MA, Cat# ab66579), 5% NGS, and 0.5% TritonX100 in 0.1MPB overnight at 4°C, washed three times for 10 min in 0.1M PB, and incubated in secondary antibody:goat anti-rabbit Alexa Fluor-488 (1:1000, ThermoFisher Scientific, Waltham, MA, Cat #A11008), 5% NGS, and 0.5% TritonX100 in 0.1M PB for 1 h at room temperature. Primary antibodies were verified by no primary control stains. Sections were mounted onto SuperFrost Plus slides (Fisher Scientific, Pittsburgh, PA) using Vectashield with DAPI hardset mounting medium (Vector Laboratories, Burlingame, CA).

### Microscopy

Fluorescent microscopy (Leica DM6000 B widefield light microscope, Leica Microsystems, Buffalo Grove, IL, USA) was used to capture color images. Positive fluorescence in images was quantified using automatic Image J (NIH) triangle thresholding feature. The implementation of the ImageJ automatic triangle algorithm has been previously described [38]. Briefly, the algorithm assumes a maximum peak near one end of the histogram and searches for intensity toward the end of the histogram bins. Three to five sections were analyzed bilaterally per animal for the dorsal dentate gyrus and values were averaged and expressed as percent positive stain. In addition, the number of TNF-α positive cells overlaid with DAPI, an indicator of cellular nuclei, in all the images taken was counted manually. All analyses including thresholding and counting were made blind to treatment conditioning. Tissue from two poor perfusions (*n* = 1 0-h saline and *n* = 1 24-h saline) that yielded high nonspecific background which interfered with thresholding was dropped from the analysis. In these perfusions, gross inspection of the brain did not reveal a fixed appearance—void of blood in circulatory system (white to pale yellow color), but instead was found to be reddish, indicative of blood present. Moreover, automatic thresholding failed on this tissue and resulted in exclusion from analysis. The decision to exclude these two samples was made blind to the treatment group. Publication images were compiled with the Adobe Photoshop CS software (Creative Cloud Photoshop v22.1, San Jose, CA, USA). Color levels and background were reduced for optimal representation with level tools. Images from all experimental groups were treated equally.

### Chronic Escalating Heroin Administration and Withdrawal

**Fig. 1:**
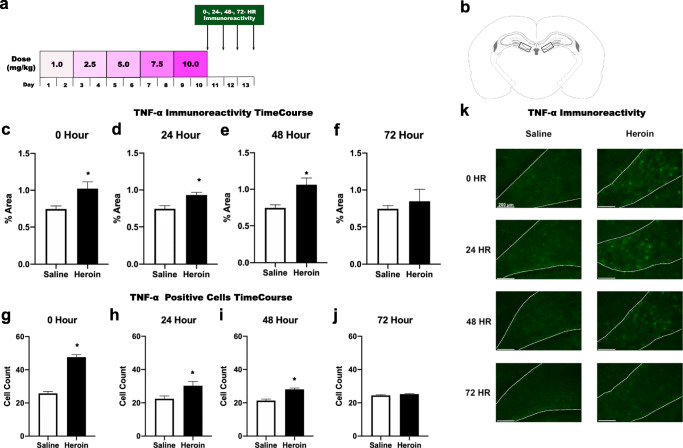
Chronic heroin administration and withdrawal increase TNF-α in the dentate gyrus of the dorsal hippocampus. Experimental timeline (**a**). Paxinos and Watson schematic depicting bilateral image acquisition location for the DG of DH (**b**). Quantification of positive fluorescence stain (Alexa-488) of TNF-α at timepoints 0 h (**c**), 24 h (**d**), 48 h (**e**), and 72 h (**f**) into withdrawal (*N* = 51, *n* = 6-8). Quantification of positive cells TNF-α at timepoints 0 h (**g**), 24 h (**h**), 48 h (**i**), and 72 h (**j**) into withdrawal (*N* = 51, *n* = 6-8). Representative images (×20) for saline and heroin animals at all timepoints taken within the DG of the DH. Dotted white lines indicate region of interest (**k**). *, statistically significant difference relative to respective control. Error bars indicate SEM

Animals were randomly assigned to drug (heroin or saline) treatment and heroin withdrawal timepoint (0, 24, 48, or 72 h) and underwent chronic escalating heroin administration as described previously [21]. Briefly, rats were injected with heroin or saline 3 times daily (subcutaneous, s.c.) over 24-h periods for 10 days, with a dose increase every other day: 3.0 (3 × 1.0) mg/kg/day on days 1-2, 7.5 (3 × 2.5) mg/kg/day on days 3-4, 15.0 (3 × 5.0) mg/kg/day on days 5-6, 22.5 (3 × 7.5) mg/kg/day on days 7-8, and 30 (3 × 10) mg/kg/day on days 9-10 (Fig. 1a). Animal weights are measured on every dose increase day and subsequent withdrawal timepoints. This chronic escalating and withdrawal paradigm has been shown to robustly produce withdrawal at the 24-h timepoint indicating both dependence of drug and subsequent withdrawal [21, 39].

### Chronic Heroin and Withdrawal-Enhanced Fear Learning

**Fig. 2:**
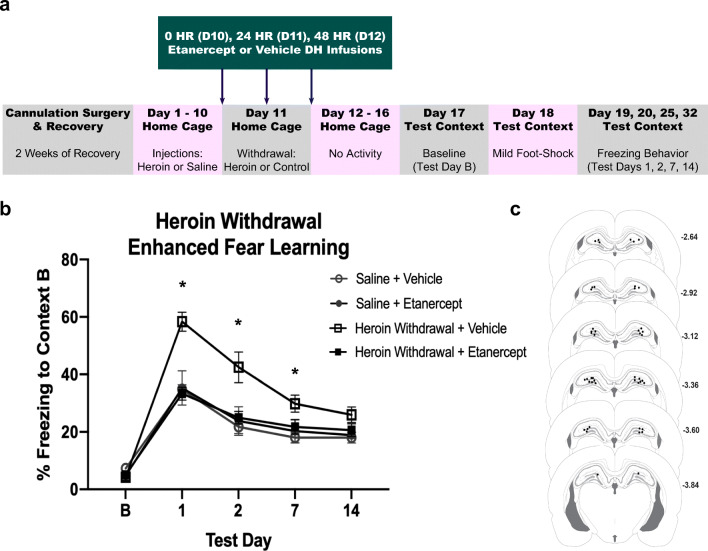
Etanercept (TNF-α inhibitor) prevents the development of heroin withdrawal-enhanced fear learning. Experimental timeline (**a**). Intra-DH etanercept infusions significantly attenuated enhanced fear learning (*N* = 31, *n* = 6-9) (**b**). Paxinos and Watson schematic with DH cannula placements shown. Each circle represents termination site of the cannula tract (**c**). *, statistically significant difference relative to respective control. Error bars indicate SEM

**Fig. 3:**
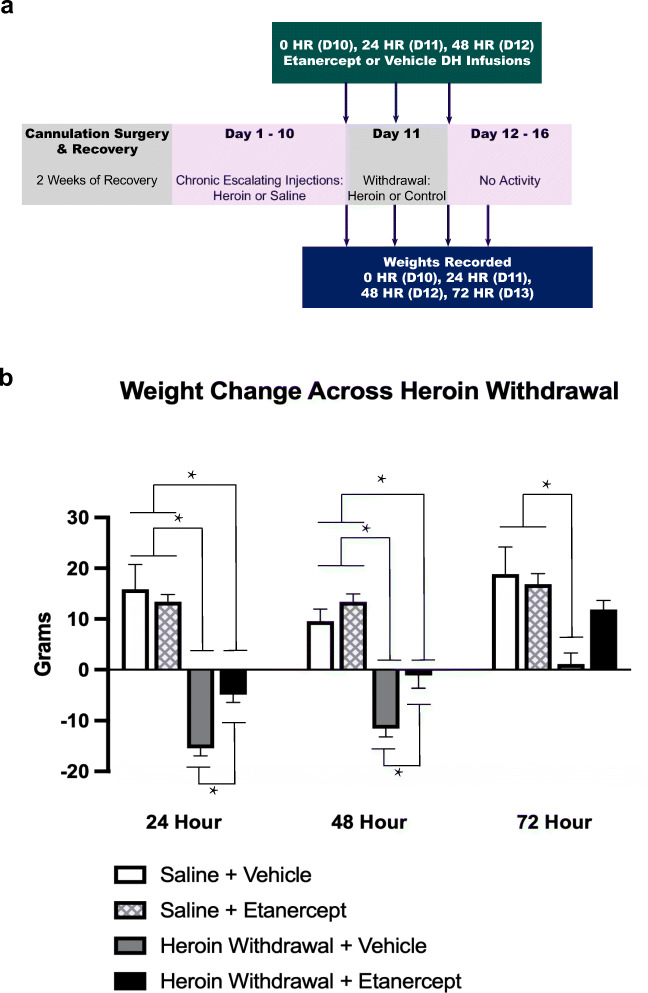
Etanercept (TNF-α inhibitor) mitigates the heroin withdrawal-induced weight loss. Experimental timeline (**a**). Intra-DH etanercept infusions significantly reduced the withdrawal-induced weight loss (*N* = 31, *n* = 6-9) (**b**). Cannula placements can be seen in Fig. 2c. *, statistically significant difference as indicated by bars. Error bars indicate SEM

This procedure has been previously described at length [21]. Briefly, animals undergo chronic escalating heroin administration and withdrawal in their home cage. Seven days after the start of withdrawal, animals were placed into a novel context for 15 min of habituation. On day 8, animals were placed into the same context for a single scrambled foot shock (1mA, 1s) at 3 min and 12 s. On days 9, 10, 15, and 22 (test days 1, 2, 7, and 14), animals are placed into the same context for 8 min and 32 s and behavior was recorded to measure freezing behavior, a measure of learned fear (Fig. 2a). The Ethovision XT video tracking software (Noldus Information Technology Inc.) was used to analyze freezing behavior. The activity analysis feature (activity threshold = 10) was used to calculate the percent of time each animal was inactive during each contextual fear test and at baseline. Weight was measured at each timepoint to determine withdrawal-induced weight change, as well as infusion-induced weight change (Fig. 3a).

### Statistical Analysis

Experiment 1 was run using separate cohorts for each of the withdrawal timepoints. Therefore, planned comparisons using unpaired, two-tailed Student’s *t* tests determine specifically whether drug treatment altered TNF-α immunoreactivity and cell counts between the 0-h, 24-h, 48-h, and 72-h timepoints. In experiment 2, a one-way (heroin, saline) ANOVA was used to analyze baseline freezing data. A 2 (heroin, saline) × 2 (etanercept, saline) × 4 (test day) repeated-measures ANOVA was used to analyze freezing behavior. A 2 (heroin, saline) × 2 (etanercept, saline) × 3 (timepoint) repeated-measures ANOVA was used to analyze weight change across withdrawal. Significant interactions for both freezing behavior and weight change were examined using Tukey’s post hoc comparisons.

## Results

### Experiment 1: Chronic Heroin Administration and Withdrawal Increase TNF-α Immunoreactivity and Positive Cells at 0-h, 24-h, and 48-h Timepoints

TNF-α immunoreactivity was significantly enhanced by chronic heroin administration and withdrawal in the DG of the DH. TNF-α immunoreactivity was increased during 0-h heroin withdrawal (*t*_(18)_ = −2.727, *p* = .029) (Fig. 1c), 24-h heroin withdrawal (*t*_(21)_ = −3.416, *p* = .003) (Fig. 1d), and 48-h heroin withdrawal (*t*_(21)_ = −3.190, *p* = .008) (Fig. 1e) relative to saline controls, but no difference was observed at 72-h withdrawal (*t*_(21)_ = −.277, *p* = −0.603) (Fig. 1f). Additionally, TNF-α positive cells were significantly enhanced by chronic heroin administration and withdrawal in the DG of the DH. TNF-α positive cells were increased during 0-h heroin withdrawal (*t*_(18)_ = 12.107, *p* < .001) (Fig. 1g), 24-h heroin withdrawal (*t*_(21)_ = −2.542, *p* = .024) (Fig. 1h), and 48-h heroin withdrawal (*t*_(21)_ = 5.651, *p* = .002) (Fig. 1i) relative to saline controls, but no difference was observed at 72-h withdrawal (*t*_(21)_ = 1.583, *p* = −0.174) (Fig. 1j). These results show that both a combination of chronic heroin administration and subsequent withdrawal is necessary for increased TNF-α immunoreactivity within the DG as TNF-α immunoreactivity was increased 0, 24, and 48 h following heroin administration and withdrawal.

### Experiment 2: Etanercept (TNF-α Inhibitor) Prevents Chronic Heroin and Withdrawal-Enhanced Fear Learning and Mitigates Withdrawal-Induced Weight Loss

Intra-DH etanercept prevented enhanced fear learning and mitigated withdrawal-induced weight loss. There was no effect of heroin treatment or etanercept infusion on baseline contextual freezing (*F*_(3,25)_ = 2.216, *p* = .111), indicating that there is no generalized fear to the novel context. A 2 × 2 × 4 repeated-measures ANOVA revealed a significant main effect of heroin treatment (*F*_(1,25)_ = 15.366, *p* < .001) and a significant main effect of etanercept treatment (*F*_(1,25)_ = 8.772, *p* = .007). These main effects of heroin treatment and etanercept treatment were on contextual freezing. There was also a significant effect of test day (*F*_(3,75)_ = 42.100, *p* < .001), indicating that conditioned freezing behavior diminished over time, thus suggesting extinction of contextual conditioning. Importantly, there was a significant heroin treatment by etanercept infusion interaction (*F*_(1,25)_ = 13.195, *p* < .001). On test days 1, 2, and 7, Tukey’s post hoc comparisons revealed heroin-withdrawn, vehicle-treated animals exhibited significantly higher freezing behavior compared to animals that were saline controls and vehicle-treated on test days 1 (*p* = .001), 2 (*p* = .005), and 7 (*p* = .042) replicating enhanced fear learning. Heroin-withdrawn, etanercept-treated animals exhibited significantly less freezing than withdrawn controls that received intra-DH vehicle on test days 1 (*p* < .001), 2 (*p* = .016), and 7 (*p* = .036). Furthermore, heroin-withdrawn, etanercept-treated animals exhibited a comparable amount of freezing behavior (no significant difference) to both saline control groups (*p* > .05) (Fig. 2b). These results indicate that intra-DH etanercept during heroin withdrawal prevented future enhanced fear learning.

A 2 × 2 × 3 repeated-measures ANOVA revealed a significant main effect of heroin treatment on withdrawal weight change (*F*_(1,25)_ = 25.410, *p* < .001). Weight loss in rodents is a hallmark symptom of opioid withdrawal [40], and may be observed due to increased diarrhea, decreased appetite, or anorexia. Importantly, there was a significant main effect of etanercept treatment on withdrawal weight change (*F*_(1,25)_ = 5.333, *p* = .029) indicating that intra-DH etanercept infusions may have prevented the heroin withdrawal decrease in weight. There was also an overall effect of withdrawal time (*F*_(2,50)_ = 41.192, *p* < .001), indicating over the course of withdrawal animals started to gain weight again (Fig. 3b).

Tukey’s post hoc comparisons revealed during the 24-h withdrawal timepoint, both the saline control groups did not differ in weight change (*p* > .05). Importantly, heroin-withdrawn, vehicle-treated animals had a significantly higher weight change compared to both the saline control groups (*p* < .001) and heroin-withdrawn, etanercept-treated animals also had a significantly higher weight change compared to both the saline control groups (*p* < .001). Strikingly, heroin-withdrawal, vehicle-treated animals had a significantly higher weight change in comparison to heroin-withdrawn, etanercept-treated animals (*p* < .05), indicating that etanercept treatment mitigated the withdrawal-induced weight loss at 24-h withdrawal (Fig. 3b).

Similarly, during the 48-h withdrawal timepoint, Tukey’s post hoc comparisons revealed both the saline control groups did not differ in weight change (*p* > .05). Importantly, heroin-withdrawn, vehicle-treated animals had a significantly higher weight change compared to both the saline control groups (*p* < .001) and heroin-withdrawn, etanercept-treated animals also had a significantly higher weight change compared to both the saline control groups (*p* < .001). Strikingly, heroin-withdrawn, vehicle-treated animals had a significantly higher weight change in comparison to heroin-withdrawn, etanercept-treated animals (*p* < .05), indicating that etanercept treatment mitigated the withdrawal-induced weight throughout the 48-h withdrawal timepoint. During the 72-h withdrawal timepoint, both the saline control groups did not differ in weight change (*p* > .05), and heroin-withdrawn, vehicle-treated animals had a significantly higher weight change compared to both the saline control groups (*p* < .001) (Fig. 3b). Overall, these results show that the etanercept was able to prevent enhanced fear learning, as well as mitigate heroin withdrawal-induced weight loss.

## Discussion

The current study demonstrates for the first time that DH TNF-α signaling mediates some long-lasting maladaptive behavioral responses induced by chronic heroin and withdrawal. We have shown that exposure to chronic heroin administration and withdrawal induces TNF-α immunoreactivity and TNF-α positive cell counts within the DG, and disrupting DH TNF-α signaling during withdrawal blocks the development of enhanced fear learning. Critically, this manipulation also mitigates heroin withdrawal-induced weight loss, a hallmark sign of withdrawal in rodent models. These findings provide new evidence that heroin withdrawal-induced TNF-α is necessary for the development of future enhanced fear learning and is a potential mechanism by which opioids and opioid withdrawal can elicit fear-related and arousal-related features of PTSD symptomatology.

The present findings suggest that neurobiological changes, specifically increases in TNF-α immunoreactivity, leave the animals hypersensitive to future stressors. Exposure to chronic heroin administration and withdrawal increases TNF-α immunoreactivity within the DG up to 48 h into withdrawal. This finding complements our recently published IL-1 findings [21]; however, IL-1 was only seen to be upregulated during the 24-h withdrawal period. The current experiments demonstrate both chronic heroin administration and withdrawal increase TNF-α immunoreactivity, as TNF-α was also increased at the non-withdrawal 0-h timepoint. This suggests TNF-α levels may elevate at some point during chronic, escalating heroin administration and persists well into the withdrawal period. This is consistent with the literature, as TNF-α is elevated following morphine use and withdrawal [41–43], naloxone-precipitated opioid withdrawal [44], and morphine withdrawal-driven synaptic plasticity [45]. These opioid-related neuroimmune alterations can lead to long-lasting neural adaptations and increase vulnerability to health detriments, such as anxiety disorders, associated with use [46–48]. The current experiments establish that TNF-α signaling is critical to the learning processes resulting in the formation of a maladaptive behavioral phenotype following heroin use and withdrawal.

The present study demonstrates that heroin and withdrawal-induced enhanced fear learning is driven by hippocampal TNF-α signaling, as inhibiting DH TNF-α signaling disrupts future exaggerated fear conditioning. A significant body of literature implicates TNF-α signaling as an integral component of the learning and memory processes. Specifically, a local increase of TNF-α in the hippocampal dentate gyrus activates TNF receptor type 1, which triggers an astrocyte-neuron signaling cascade resulting in persistent functional modification of hippocampal excitatory synapses [33]. Moreover, TNF-α modulates hippocampal synapses via AMPA receptor trafficking and GABA_A_ receptors [49, 50]. This suggests that TNF-α is a key physiological regulator of hippocampal synaptic activity and excessive TNF-α may alter synaptic functioning and learning mechanisms, potentially impacting the development of enhanced fear learning. Specifically, the current studies suggest that blocking the TNF-α increase reduced conditioned freezing behaviors, suggesting an attenuation of enhanced fear learning. Consistent with this, studies have shown that increases in TNF-α are necessary for sustained fear learning, both in cued and contextual acquisition and extinction [33, 51], and inhibiting TNF-α reduces memory deterioration induced by LPS [32]. These studies as well as the present work suggest that TNF-α signaling plays a critical role in learning and memory processes.

The focus of the current work examined the role of hippocampal TNF-α in heroin withdrawal for enhanced fear learning and weight loss, and directly compliments our previous work indicating that IL-1 signaling is involved in heroin withdrawal and enhanced fear learning [21]. Evidence suggests that IL-1β’s effects may be a result of synergistic interaction with TNF-α [52]. The interdependent relationship between IL-1β and TNF-α has been demonstrated in multiple models, such that TNF-α action promotes additional TNF-α transcription, as well as the production and release of IL-1β [53, 54]. Additionally, both the TNF-α and IL-1 signaling pathways lead to the activation of the proinflammatory transcription factor, NF-κB [55–57], suggesting that these cytokines work together in multiple neuroinflammatory processes. Studies to examine the interaction between IL-1 and TNF-α signaling in heroin withdrawal-enhanced fear learning would provide more information regarding the long-lasting neuroimmune adaptations driving this behavioral effect.

Evidence suggests that glial activation leads to the release of proinflammatory molecules that modulate neuronal activity crucial to the complex syndrome of opioid dependence and withdrawal. In particular, glial cells appear to be primarily responsible for TNF-α release [58], which may be mediated through glutamate release from astrocytes and microglia [59]. Studies have shown that astrocyte-derived TNF-α may alter hippocampal synapses and modify excitatory synapses [33], while microglial production of TNF-α has been implicated as a key element of sustained fear memory [51]. Additionally, high levels of TNF-α can signal astrocytes and microglia via activation of NF-κB to elicit a proinflammatory response which increases production of a large number of other cytokines, such as IL-1 [57, 60, 61]. Cell-specific studies on both microglia and astrocytes may increase our understanding to the origin of TNF-α in our model, as well as its mechanism in heroin use and withdrawal to alter future learning and memory processes. Future studies can extend this line of work to examine the cellular source and targets of this TNF-α signaling, as well as the role of glia in modulating exaggerated fear learning responses.

The present work focused on the DH, a brain region critical to learning and memory processes. As disruption of TNF-α signaling mitigated withdrawal-induced weight loss, future studies should investigate TNF-α expression in other areas involved in opioid abuse, such as the ventral tegmental area, locus coeruleus, or periaqueductal gray (PAG). Specifically, the PAG can be targeted to better understand the effects of TNF-α in heroin withdrawal and withdrawal symptomology. Functional studies have implicated the PAG in the expression of symptoms of opioid withdrawal, but the molecular mechanisms involved are not fully understood. Further studies should investigate the role of TNF-α expression in other brain regions involved in opioid abuse, such as the PAG, that may also contribute to enhance fear learning and alter heroin withdrawal-induced weight loss or other withdrawal-related behaviors.

Strikingly, intra-DH etanercept infusions mitigated heroin withdrawal-induced weight loss. This intriguing finding demonstrates a role for DH TNF-α signaling in both learning processes and withdrawal behaviors, such as weight loss. Although this study evaluated weight loss and freezing behavior separately, it would be interesting to study the correlation between these two behaviors. As regards to weight loss, specifically, we believe TNF-α may be exerting an anorexic effect in opposition to ghrelin. Studies have shown that lower levels of total TNF-α promoter methylation had higher success with a weight loss program [62], and therefore the dysregulation of TNF-α following heroin use and withdrawal may contribute to the weight loss associated with withdrawal. Although the current study focused solely on weight loss, as it is a prominent symptom of rodent opioid withdrawal, it would be interesting to investigate TNF-α’s effect on other withdrawal behaviors such as wet dog shakes, teeth chattering, and diarrhea. It is possible that these behaviors may also be mediated by TNF-α signaling, as it has been previously shown that downregulating TNF-α signaling in the periaqueductal gray (PAG) of mice decreased the physical symptoms of morphine withdrawal [63]. In particular, studies have shown that heroin administration can directly stimulate glial toll-like receptor 4 (TLR4) that results in the overexpression of TNF-α and injection of TNF-α into the PAG produces withdrawal symptoms [64]. This suggests TNF-α regulates symptoms of opioid withdrawal and inhibition can mitigate these effects. Further studies can investigate the effect of region-specific infusions of etanercept on other symptoms of opioid withdrawal. In line with our current findings, we hypothesize that systemic etanercept or peripheral administration would decrease other behaviors of heroin withdrawal. Unfortunately, biologic TNF inhibitors, such as etanercept, do not cross the blood-brain barrier (BBB) [65]. TNF inhibitors have been engineered to penetrate the BBB in combination with a transgenic mouse line through the fusion of extracellular domain of the type II human TNF receptor to a chimeric monoclonal antibody designed to function as a fusion protein [66]. Further studies can study the peripheral effect of TNF inhibitors on heroin withdrawal behaviors using the novel engineered TNF inhibitors. We hypothesize that given systemically, novel engineered TNF inhibitors may ameliorate other physical symptoms of withdrawal as the delivered inhibitor will reach multiple brain regions as well as peripheral processes.

In summary, our exciting findings demonstrate that heroin use and withdrawal increase expression of hippocampal TNF-α, and inhibition of TNF-α signaling disrupts future enhanced fear learning, as well as mitigates heroin withdrawal-induced weight loss. Collectively, these data provide important new evidence that chronic heroin administration and withdrawal alter hippocampal neuroimmune signaling and downstream behavioral responses, such as enhanced susceptibility to future stressors and withdrawal-induced weight loss. The current study identifies neuroimmune targets that can be used to alleviate long-term maladaptive responses stemming from a history of chronic heroin use and protracted withdrawal.

## Data Availability

The datasets that support the findings of this study are available from the corresponding author upon reasonable request.

## Declarations

### Animal Experiments

All procedures were conducted in accordance with the National Institutes of Health guide for care and use of laboratory animals and with approval from the University of North Carolina at Chapel Hill Institutional Animal Care and Use Committee. All efforts were made to minimize animal suffering, reduce the number of animals used, and utilize alternatives to in vivo techniques, when available.
